# Passing of the baton 2022

**DOI:** 10.1002/acn3.51703

**Published:** 2022-12-03

**Authors:** Jack Kessler

**Affiliations:** ^1^ Neurology Northwestern University Chicago Illinois USA

In 2013, the ANA launched a new open‐access journal, the *Annals of Clinical and Translational Neurology*, and I had the privilege of being the first editor‐in‐chief. The beginning of the journal is so fresh in my mind that it is amazing that more than 9 years have passed, and that the time has come to pass the baton to a new EIC. In an subsequent editorial the next EIC, Ahmet Hoke, will introduce himself and outline his vision and plans for the future of the journal.

Like all new ventures, the beginnings for ACTN were uncertain with no guarantee of success either academically or fiscally. Convincing authors to submit strong manuscripts to an unknown journal with an unclear future was difficult. But *ACTN* was blessed with the imprimatur and prestige of the ANA, the support of its sister journal, the *Annals of Neurology*, and an exceptional group of founding associate editors who built the journal. I would like to thank the first group of editors, Flint Beal, Andrew Cole, Anne Cross, Leon Epstein, Ahmet Hoke, and Steven Warach, for their efforts in making ACTN a success. *ACTN* also was fortunate to be able to attract Emmanuelle Waubant, Puneet Opal, Jennifer Graves, Raquel Sanchez‐Valle, and Sean Savitz as editors who maintained the editorial excellence established at the onset of the journal.

*ACTN* is now a well‐established and recognized journal that helps to position the ANA well for the open‐access future of publishing. Its success reflects the support and contributions of many people to whom I am grateful. First, I would like to thank the ANA Board and Eva Feldman, the President when ACTN was conceptualized, for giving me the opportunity to be the EIC. I have been fortunate that there has been unwavering support from both the ANA Board and our Editorial Board over the past 9 years. I also would like to thank Clif Saper, and more recently Ken Tyler, for their help and insights as EIC of the *Annals of Neurology* since the relationship between the two journals was an instrumental part of the success of ACTN. In 2016 a unique and now popular interactive clinical case series, InterACTN, was started with Danny Bega as the editor, and I believe that InterACTN's success will lead to additional interactive features in the coming years. We also have been fortunate to have the support and expertise of our publisher, Cathy Krendel, and the staff at John Wiley and Sons as well as our assistant editors, Emily Hammond and Keegan Brewster. ACTN also benefited greatly from the expertise of Kathleen Gaffney, an outside editorial consultant who helped negotiate our new publishing contract, and of Morna Conway who coordinated the selection of a new EIC and who is overseeing the transition. And I am deeply grateful to the cadre of Reviewers who helped to assure the quality of what we publish, and I am particularly indebted to those Reviewers who helped before ACTN was well recognized by the neurological community.

My experiences as EIC have been exciting, complicated, arduous, sometimes frustrating, occasionally painful, but always personally rewarding. I am very fortunate to have had the opportunity to help establish the journal. I am deeply invested emotionally in ACTN's success, and it was very important to me for the ANA to appoint a new EIC who can continue to expand the footprint and impact of the journal. I am delighted that someone as outstanding as Ahmet Hoke was chosen to lead ACTN into the future.

